# Supplementary material for: Gait, physical activity and tibiofemoral cartilage damage: a longitudinal machine learning analysis in the Multicenter Osteoarthritis Study
Source: Br J Sports Med. Author manuscript; Available in PMC 2023 Sep 28. (PMC10423491; doi:10.1136/bjsports-2022-106142)
Supplement: 1 [file NIHMS1920412-supplement-1.pdf]

Supplement for:

Gait, physical activity, and tibiofemoral cartilage damage: A longitudinal machine learning analysis in the Multicenter Osteoarthritis Study

Kerry E. Costello[1,2,3], David T. Felson[2], S. Reza Jafarzadeh[2], Ali Guermazi[2], Frank W. Roemer[2,4], Neil A. Segal[5,6], Cora E. Lewis[7], Michael C. Nevitt[8], Cara L. Lewis[1,2], Vijaya B. Kolachalama[1,2], Deepak Kumar[1,2]

[1] Boston University, Boston, MA, USA
[2] Boston University School of Medicine, Boston, MA, USA
[3] University of Florida, Gainesville, FL, USA
[4] University of Erlangen, Erlangen, Germany
[5] University of Kansas Medical Center, Kansas City, KS, USA
[6] The University of Iowa, Iowa City, IA, USA
[7] University of Alabama at Birmingham, Birmingham, AL, USA
[8] University of California at San Francisco, San Francisco, CA, USA

Corresponding author:

Deepak Kumar, PT, Ph.D.
635 Commonwealth Avenue, Boston, MA 02215
kumard@bu.edu

Sensitivity analysis examining subsample with baseline cartilage damage

In the subsample of knees with baseline cartilage damage (Table S1), 26% had cartilage worsening at 2-year follow-up. For each predictor, we calculated the marginal causal risk difference of each category of the predictor on cartilage worsening, compared to the corresponding reference category using g-computation. Continuous variables were categorized using tertile cutpoints calculated from the full sample (as detailed in the main manuscript). The models included the same predictors as in the main manuscript except for baseline cartilage damage (i.e., 9 total predictors included).

As in the main analysis, in the subsample with baseline cartilage damage, the g-computation analysis identified an increased risk of cartilage worsening for individuals with KLG 2 versus 0 (17.9% per 100 individuals) and for pain during walking of mild versus none (16.3% per 100 individuals) (Figure S1). In the main analysis a lateral ground reaction force (GRF) impulse of 1.8 N*s or higher compared to <1.1 N*s had a higher risk of cartilage worsening. In the subsample, the point estimate was similar to the main analysis (6.1% versus 7.2% per 100 individuals), but the 95% confidence interval included zero. Similarly, point estimates were similar for the middle versus lowest tertile of time spent lying (7.1% versus 5.4% per 100 individuals) and for the highest versus lowest tertile of maximum vertical GRF unloading rate (7.8% versus 6.6%) but both 95% confidence intervals included zero. These wider confidence

intervals could be related to the smaller sample size of this subsample or heterogeneity within the subsample.

Table S1. Baseline demographics and clinical characteristics for subsample with baseline cartilage damage

**Feature**	**Frequency, n (%)**		**Mean ± SD**
n participants	371		
Sex:			
Female	183 (49.3%)		
Race:			
American Indian or Alaskan Native	1 (0.3%)		
Asian	3 (0.8%)		
Black or African American	36 (9.7%)		
Don't know/Refused	0 (0.0%)		
More than one race	3 (0.8%)		
Other	3 (0.8%)		
White or Caucasian	325 (87.6%)		
Clinic Site:			
University of Iowa	254 (66.0%)		
Cohort:			
New	268 (72.2%)		
Previous injury/surgery:			
Yes	89 (32.8%)		
Age (years)			61.2 ± 8.6
Body Mass Index (kg/m^2)			28.2 ± 4.8
Center for Epidemiologic Studies Depression score (/60)			5.3 ± 5.4
Hip-knee-ankle alignment (degrees, negative values indicate varus alignment)			-1.9 ± 2.7
	Study knee	*Contralateral*	
WOMAC pain during walking:			
None	293 (79.0%)	294 (79.2%)	
Mild	62 (16.7%)	62 (16.7%)	
Moderate or higher	16 (4.3%)	15 (4.0%)	
Kellgren-Lawrence Grade (KLG):			
KLG = 0	156 (42.0%)	171 (46.1%)	
KLG = 1	136 (36.7%)	130 (35.0%)	
KLG = 2	79 (21.3%)	70 (18.9%)	

SD = standard deviation; WOMAC = Western Ontario and McMaster Universities Osteoarthritis Index

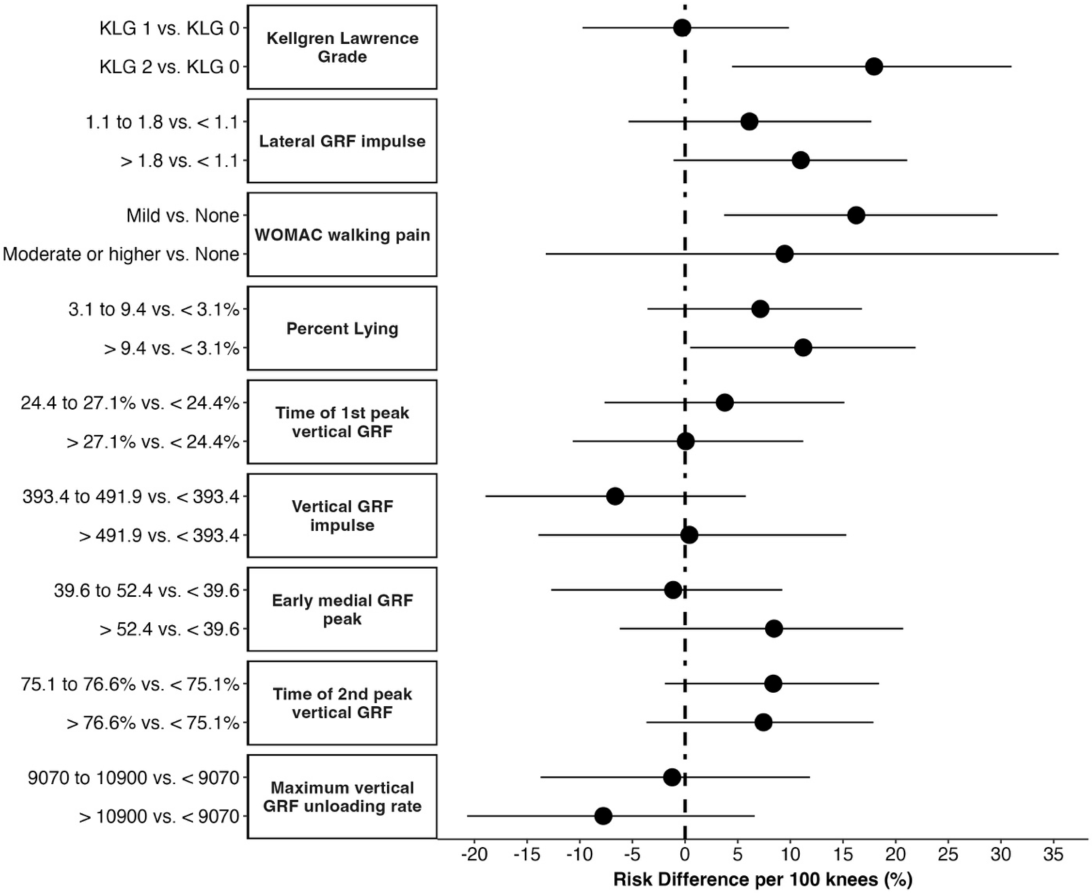

Figure S1. Causal risk differences in the subsample with baseline cartilage damage for influential predictors identified from the machine learning model
